# The Social Context of Pregnancy, Respectful Maternity Care, Biomarkers of Weathering, and Postpartum Mental Health Inequities: A Scoping Review

**DOI:** 10.3390/ijerph21040480

**Published:** 2024-04-15

**Authors:** Bridget Basile-Ibrahim, Joan Combellick, Thomas L. Mead, Alee Sorensen, Janene Batten, Robyn Schafer

**Affiliations:** 1 School of Nursing, Yale University, Orange, CT 06477, USA; joan.combellick@yale.edu (J.C.);; 2 Biomedical Libraries, Dartmouth College, Hanover, NH 03755, USA; thomas.l.mead@dartmouth.edu; 3 Harvey Cushing/John Hay Whitney Medical Library, Yale University, New Haven, CT 06510, USA; janene.batten@yale.edu; 4 Division of Advanced Nursing Practice, School of Nursing, Rutgers University, Newark, NJ 07107, USA; robyn.schafer@rutgers.edu; 5 Department of Obstetrics, Gynecology, and Reproductive Sciences, Robert Wood Johnson Medical School, Rutgers University, New Brunswick, NJ 08901, USA

**Keywords:** health equity, maternal mortality, maternal morbidity, perinatal mental health, weathering, social determinants of health, respectful maternity care

## Abstract

**Background:** Mental health disorders are the number one cause of maternal mortality and a significant maternal morbidity. This scoping review sought to understand the associations between social context and experiences during pregnancy and birth, biological indicators of stress and weathering, and perinatal mood and anxiety disorders (PMADs). **Methods:** A scoping review was performed using PRISMA-ScR guidance and JBI scoping review methodology. The search was conducted in OVID Medline and Embase. **Results:** This review identified 74 eligible English-language peer-reviewed original research articles. A majority of studies reported significant associations between social context, negative and stressful experiences in the prenatal period, and a higher incidence of diagnosis and symptoms of PMADs. Included studies reported significant associations between postpartum depression and prenatal stressors (n = 17), socioeconomic disadvantage (n = 14), negative birth experiences (n = 9), obstetric violence (n = 3), and mistreatment by maternity care providers (n = 3). Birth-related post-traumatic stress disorder (PTSD) was positively associated with negative birth experiences (n = 11), obstetric violence (n = 1), mistreatment by the maternity care team (n = 1), socioeconomic disadvantage (n = 2), and prenatal stress (n = 1); and inverse association with supportiveness of the maternity care team (n = 5) and presence of a birth companion or doula (n = 4). Postpartum anxiety was significantly associated with negative birth experiences (n = 2) and prenatal stress (n = 3). Findings related to associations between biomarkers of stress and weathering, perinatal exposures, and PMADs (n = 14) had mixed significance. **Conclusions:** Postpartum mental health outcomes are linked with the prenatal social context and interactions with the maternity care team during pregnancy and birth. Respectful maternity care has the potential to reduce adverse postpartum mental health outcomes, especially for persons affected by systemic oppression.

## 1. Introduction

Mental health conditions are the leading cause of maternal mortality in the United States (U.S.) [1]. Each year in the U.S., hundreds of thousands of women and childbearing people and their families are affected by life-altering perinatal mood and anxiety disorders (PMADs). PMADs include postpartum depression (PPD), postpartum anxiety, and birth-related posttraumatic stress disorder (PTSD) [2]. The financial impact is also significant; untreated PMADs are estimated to cost USD 14 billion per year [3,4]. Postpartum depression (PPD) alone is estimated to impact 1 in every 5 childbearing women worldwide [5]. PPD creates an environment that is not conducive to parental-role development or optimal child development [6], and severe PPD substantially raises the risk for adverse child developmental outcomes [7]. Despite the high incidence and large impact of PMADs [5], little has been documented about social and systemic factors influencing maternal mental health [8].

Perinatal mental health complications have been linked to various life-experiences in the perinatal period. For example, postpartum psychological trauma and post-traumatic stress symptoms from negative birth experiences adversely impact the lives of childbearing women and their infants by disrupting breastfeeding and mother–infant bonding [9]. These disruptions can lead to long-term health complications for both mother and child [10,11,12,13,14]. Similarly, prenatal stress is associated with negative pregnancy outcomes such as preterm birth and fetal growth restriction [15,16] and PPD [17]. Perinatal experiences of discrimination are associated with high levels of maternal stress and adverse birth outcomes for both mother and child [6,7,18,19]. In addition to being a risk factor for mistreatment and a significant stressor, socioeconomic disadvantage is also linked with negative birth outcomes and infant mortality [20,21].

Childbearing women and people from communities that have been historically marginalized in the U.S. (i.e., Black and Indigenous persons, and individuals with low socioeconomic status) have inequitably high rates of PPD [22,23]. Women and birthing people who are marginalized due to ethnicity or income are also less likely to seek care for perinatal mental health symptoms [15]. Marginalized individuals report higher rates of care that is discriminatory [24], infringes on their autonomy, and is disrespectful [25,26,27,28,29]. Additionally, individuals from marginalized communities report higher rates of mistreatment by maternity care providers and personnel [25,30,31]. A recent U.S. study found that one in five (20% of 2402 respondents) postpartum mothers reported mistreatment during their maternity care [32]. Rates of mistreatment were higher for Black, Hispanic, and multiracial mothers (30%), and for those with Medicaid (U.S. publicly funded health insurance for individuals with low household income; 30%) [32]. Negative experiences of perinatal care can have long-term consequences on maternal/child health. In contrast, positive care experiences such as respectful, person-centered maternity care has been shown to improve birth outcomes [33], increased patient satisfaction, fewer maternal and newborn complications, and lower incidence of PPD [19].

Researchers have proposed that experiences of stress, discrimination, mistreatment, and adverse social determinants of health create a “weathering” effect on Black women and other marginalized individuals [34]. Weathering is defined as increased wear and tear on the body due to exposure to a lifetime of chronic stress, mistreatment, and discrimination [34,35]. The weathering effect places marginalized women/childbearing people and their infants at an increased intergenerational vulnerability for adverse birth outcomes [35,36,37,38,39,40,41,42,43,44,45]. Weathering has been implicated as a key contributor to health disparities [46]. Biomarkers of weathering, such as advanced epigenetic age [47], telomere length [48,49], and allostatic load [35,50,51,52], are indicators of how chronic stress and discrimination get “under the skin” [52] and affect childbearing people at a cellular or biological level [34,46,47,48,49,50,51].

The purpose of this scoping review is to synthesize the current body of research regarding associations between PMADs and (1) perinatal experiences and exposures (specifically, prenatal social context and experiences of care during pregnancy and birth); and (2) biomarkers of weathering. To meet this aim, we searched for studies reporting investigations across three distinct areas of interest: (a) exposures related to social context/experiences during pregnancy and birth, (b) perinatal biomarkers of stress and weathering, and (c) PMADs outcomes.

## 2. Materials and Methods

Our study methods were guided by the JBI (formerly known as the Joanna Briggs Institute) updated methodological guidance for scoping reviews [53] and the PRISMA extension for scoping reviews (PRISMA-ScR) [54]. We registered our scoping review protocol with OSF in October 2023 (link in the Supplementary Materials).

### 2.1. Search Strategy and Procedure

A literature search was conducted to identify studies that investigated PMADs and their associations with (1) social context and life experiences during pregnancy and birth, and/or (2) physiologic markers of stress and weathering. The search strategy was designed with the guidance of two expert medical librarians (JB and TM). Databases searched included OVID Medline ALL (1946 to 11 October 2023) and OVID EMBASE (1974 to 13 October 2023). The search terms included using both controlled vocabulary and synonymous free text to capture the concepts related to the outcomes. For PMADs, search terms included *postpartum depression* or *postpartum anxiety or posttraumatic stress disorder.* Relevant exposures/experiences used the search terms *trauma*, *obstetric violence*, *discrimination*, *socioeconomic deprivation*, *socioeconomic disadvantage*, and *marginalization*. Biomarker search terms were *hair cortisol*, *inflammation*, *weathering*, *epigenetic age*, and *telomere length*. Search strategies were adjusted for syntax appropriate for each database. Electronic searches were limited to humans and the English language. Searches were completed on 16 October 2023. Supplementary efforts to identify studies included checking reference lists. The full search strategy for each electronic database is included in the Supplementary Materials. Results were uploaded into EndNote citation management software (Thompson Reuters, version 20) and deduplicated with the final set uploaded into Covidence systematic review software (Veritas Health Information, www.covidence.org accessed on 4 April 2024).

### 2.2. Inclusion Criteria

In order to compare among relatively similar settings, we included English-language publications reporting peer-reviewed original research conducted in the 20 countries with the highest gross domestic product (GDP) per capita among members of the Organization for Economic Cooperation and Development (OECD) [55]. To ensure a comprehensive scoping review, we made two exceptions to geographic setting that expanded beyond the OECD 20 highest-GDP countries. First, while the specific term “obstetric violence” has been in existence for some time [56,57], it has only more recently been adopted in the research vernacular in the highest income, English-speaking countries, and is still not widely used [58]. Because the specific term was so pertinent to this review, we did not exclude studies based on setting that specifically explored obstetric violence and mistreatment if they were set in other high-development countries where the term has been in use for a longer period of time (i.e., Russia, Croatia, Spain, and Brazil). Second, because biomarkers of stress and weathering in the perinatal period are relatively new topics in the literature, all studies that explored associations between a biologic marker of stress and weathering and an exposure or PMAD were included, regardless of geographic location. Studies were excluded if data were collected prior to 2000 (regardless of publication date), or if no measure for socioeconomic disadvantage was provided beyond stratification by household income. Only original peer-reviewed research was included; all other publication types (i.e., conference proceedings, abstracts, reviews, and commentaries) were excluded.

### 2.3. Study Selection

**Figure 1:**
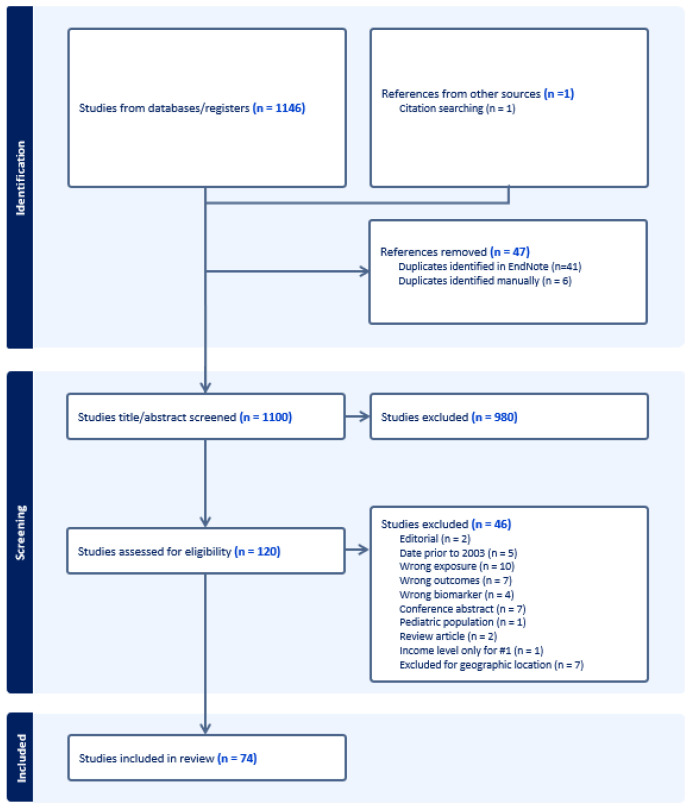
PRISMA.

The initial search resulted in 1146 records. After removing duplicates, 1099 remained. Through hand-searching of citations and recent citation alerts, one additional study was identified for screening, resulting in a total of 1100 records. Two independent, interdisciplinary reviewers (BB and RS) conducted the review of records. Discrepancies were resolved by discussion between the two reviewers to reach consensus. After title and abstract screening, 120 publications met criteria for full text review. During full text review, 46 publications were excluded, resulting in 74 studies included in this scoping review (Figure 1). Quality appraisal was not completed, as this is not in line with the study methodology [53].

### 2.4. Data Extraction

The lead author (BB) extracted data from each included study using a data extraction matrix created specifically for this review. Data extracted included: first author, year of publication, setting, sample size, study design, exposures and measurement tool for each exposure, biomarkers studied and their tissue source, PMAD outcome and measurement tool, and results. A second reviewer (AS) validated the data extraction.

## 3. Results

Seventy-four studies published between 2004 and 2023 were included in this scoping review (Table 1 and Table 2). Studies took place across a variety of regions (see Supplementary Table S1) with the majority set in the Americas (n = 34), followed by Europe (n = 28), Asia (n = 7) and Oceania (n = 5). Study designs included prospective longitudinal (n = 38), retrospective (n = 16), cohort (n = 6), and cross-sectional (n = 14) studies. The most commonly evaluated PMAD was postpartum depression (PPD; n = 62), followed by birth-related posttraumatic stress disorder (PTSD; n = 18). Five studies evaluated postpartum anxiety, and one study evaluated a researcher-defined outcome of maternal mental health status [59]. Several studies (n = 21) reported findings related to multiple exposures and/or PMAD outcomes.

Fourteen studies included perinatal measurement of biological markers of stress or weathering and our exposures or outcomes of interest (Table 2). Most studies evaluating the associations between PMADs and biomarkers of stress and weathering investigated associations with PPD (n = 12). PPD was operationalized using the Edinburgh Postnatal Depression Scale (EPDS) score in all 12 studies. The most common biomarker explored was inflammatory cytokines (n = 7) [119,120,121,123,128,129,130], followed by hair cortisol (n = 2) [122,131], maternal epigenetic age (n = 2) [125,126], maternal DNA methylation (n = 1) [127], allostatic load (n = 1) [118], and telomere length (n = 1) [124]. Four studies reported associations across all three areas of interest in this scoping review: exposure/experience, biological measure, and PMAD outcome [118,122,124,131].

### 3.1. Measurement of Exposures/Experiences during Pregnancy and Birth

Most of the measurements for the social context exposures/experiences independent variables were obtained through participant self-reports. Validated measurement tools used to measure the independent exposure/experiences in the included studies are listed in Table 3.

### 3.2. Associations between PMADs and Social Exposures

**Figure 2:**
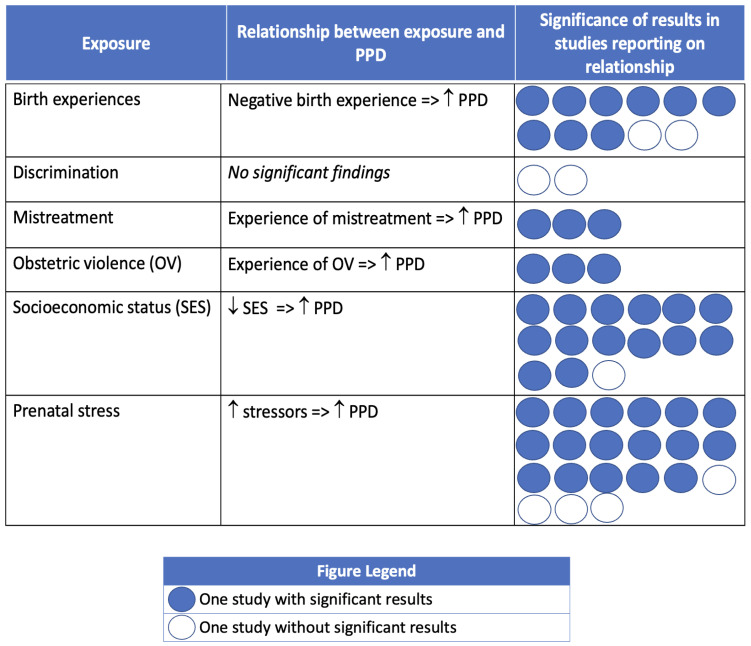
Postpartum Depression (PPD) and social context of pregnancy and birth. Note: Circles are used to represent an included study reporting on the association of the PMAD outcome with the exposure of interest. Dark circles indicate that the study found a significant association between the exposure and PMAD. Empty circles indicate that the study did not have significant findings regarding the exposure and PMAD.

**Figure 3:**
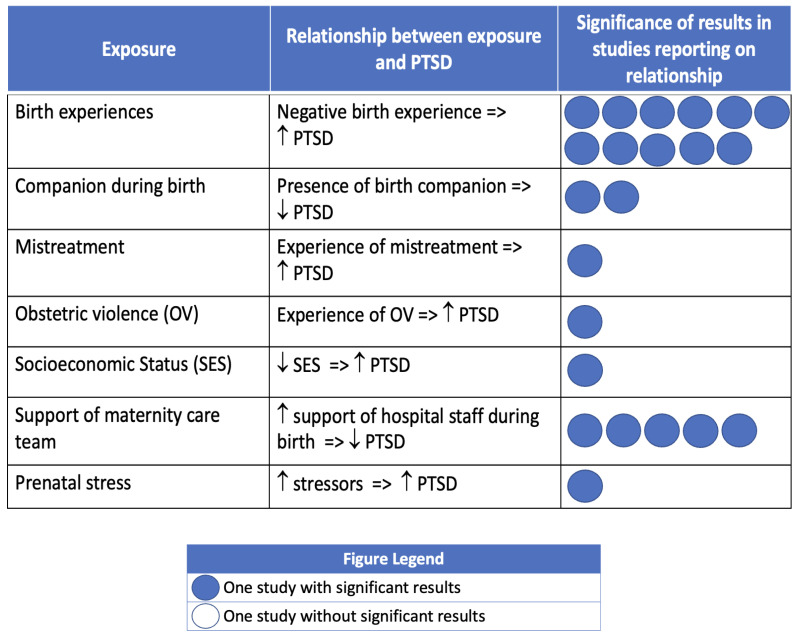
Birth-related post-traumatic stress disorder (PTSD) and social context of pregnancy. Note: Circles are used to represent an included study reporting on the association of the PMAD outcome with the exposure of interest. Dark circles indicate that the study found a significant association between the exposure and PMAD. Empty circles indicate that the study did not have significant findings regarding the exposure and PMAD.

**Figure 4:**
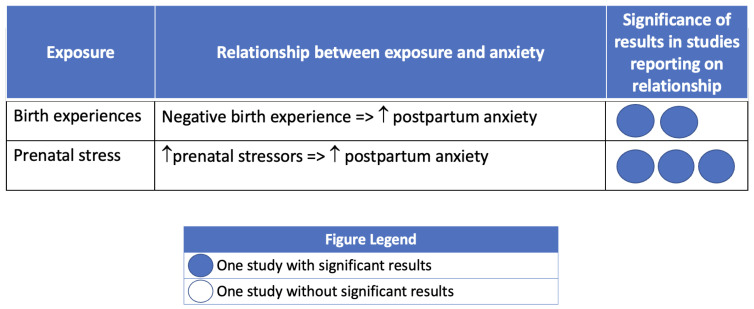
Postpartum anxiety and social context of pregnancy. Note: Circles are used to represent an included study reporting on the association of the PMAD outcome with the exposure of interest. Dark circles indicate that the study found a significant association between the exposure and PMAD. Empty circles indicate that the study did not have significant findings regarding the exposure and PMAD.

Most studies that investigated associations between social context and PMADs reported significant associations between at least one exposure and PPD, postpartum anxiety, or birth-related PTSD. Figure 2, Figure 3 and Figure 4 summarize the findings, organized by PMAD outcome.

### 3.3. Postpartum Depression

Postpartum depression (PPD) was the most frequently investigated PMAD (n= 62). The Edinburgh Postnatal Depression Scale (EPDS) [132] was the most commonly used measure of postpartum depression (n = 48). Severity of PPD symptoms was assessed by raw score, with a cutoff score (most often ≥10) to indicate a diagnosis of PPD.

In 9 of the 11 studies exploring associations with birth experiences, having a negative birth experience significantly increased the likelihood of PPD symptoms (n = 4) and diagnosis (n = 5). Experiencing mistreatment by maternity care personnel was significantly associated with increased PPD symptoms (n = 1) [101] and diagnosis (n = 2) [89,108]. Similarly, experiencing obstetric violence was associated with a greater likelihood of PPD diagnosis (n = 3) [93,110,113]. Fourteen out of fifteen studies found that PPD symptoms (n = 9) and diagnosis (n = 5) significantly increased with increasing levels of socioeconomic disadvantage. Two studies evaluated discrimination and PPD, but neither reported significant associations [92,124]. Stress in the form of daily stressors and/or stressful life events was the most commonly investigated exposure in relationship to PPD (n = 21). Seventeen studies found that as prenatal stress increased, symptoms (n = 7) and diagnosis (n = 10) of PPD significantly increased.

### 3.4. Birth-Related Post-Traumatic Stress Disorder

Eighteen studies evaluated relationships between exposures of interest and birth-related PTSD. Birth-related PTSD was most commonly measured by the City Birth Trauma Scale (CBTS; n = 5) [133] and the Impact of Event Scale (IES; n = 5) [134,135]. The CBTS was published in 2018, thus, only the more recent studies have used this measure.

The two most investigated exposures related to birth-related PTSD were birth experiences (n = 11) and support from the maternity care team during labor and birth (n = 5). A negative birth experience was significantly associated with increased PTSD symptoms (n = 5) and diagnosis (n = 6). Participants who felt more supported during labor and birth by their maternity care team reported significantly decreased PTSD symptoms (n = 5). Similarly, the presence of a companion or doula during labor and birth was associated with significantly fewer symptoms of PTSD (n = 4) [75,111,113,117]. The influence of mistreatment and obstetric violence on PTSD were investigated in one study each [89,117]. Experiencing mistreatment from the healthcare team was significantly associated with an increased likelihood of PTSD diagnosis [89], while experiencing obstetric violence was associated with a significant increase in PTSD symptoms [117]. One study reported that as socioeconomic status decreased, PTSD diagnosis significantly increased [67]. One additional study reported on the association between prenatal stress and PTSD, and found that as stress increased, PTSD symptoms significantly increased [68].

### 3.5. Postpartum Anxiety

Five studies evaluated associations between exposures of interest and postpartum anxiety. Postpartum anxiety was most commonly measured using the State-Trait Anxiety Inventory (STAI; n = 3) [136]. Participants who reported a negative birth experience were more likely to have postpartum anxiety in two studies [60,106]. Three studies found a significant relationship between prenatal stress and postpartum anxiety [68,72,105].

### 3.6. Studies including Biomarkers of Stress and Weathering

**Figure 5:**
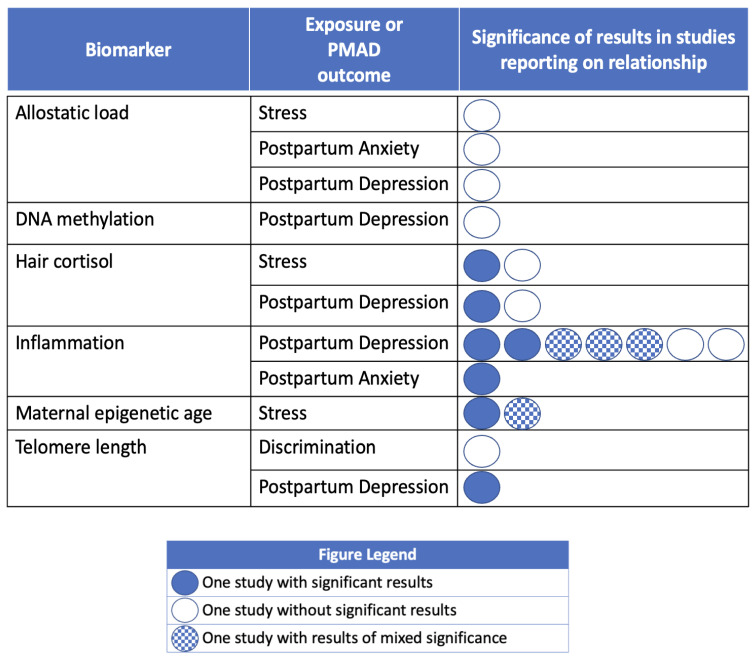
Evidence for associations between biomarkers of stress and weathering and social context/maternal mental health outcomes. Note: Circles are used to represent an individual included study reporting on the association of the PMAD outcome with the exposure of interest. Dark circles indicate that the study found a significant association between the exposure and PMAD. Empty circles indicate that the study did not have significant findings regarding the exposure/PMAD and the biomarker of interest.

Our search identified 14 studies that evaluated relationships between biological measures of stress and weathering and our exposures and/or outcomes of interest (Table 2). A portion of the included studies did not report any significant findings, and another set had conflicting results and/or minimal results of significance supporting the associations explored (Figure 5). Biomarkers investigated included: allostatic load, DNA methylation, hair cortisol, inflammatory markers/cytokines, telomere length, and maternal epigenetic age.

Allostatic load and DNA methylation did not have a significant association with PMADs or our exposures of interest. One study explored allostatic load in a US sample of 845 individuals, and did not find significant associations between allostatic load and stress, PPD, nor postpartum anxiety [118]. Similarly, there was no evidence of a significant relationship between DNA methylation and PPD in one study exploring this biomarker in 36 Japanese individuals [127].

This review identified conflicting evidence in research exploring an associations with hair cortisol. In a prospective longitudinal study of 44 women in Southern Spain, Caparros-Gonzalez and colleagues found that first and third trimester hair cortisol levels were significantly associated with PPD symptoms [122]. In contrast, among 196 individuals in Germany, Stickel and colleagues did not find a significant relationship between hair cortisol and prenatal stress, nor between hair cortisol and PPD [131].

Biological indicators of inflammation were explored in seven studies, with mixed results. Two research teams found an inverse association between levels of anti-inflammatory cytokines (i.e., IL-4, IL-6, IL-10) and PPD symptoms [128,130]. Two studies found an inverse association between PPD symptoms and TNFα (a proinflammatory marker), but no significant associations among the other inflammatory markers investigated [121,123]. In a Thai study with 71 participants, Roomruangwong and colleagues found increased C-reactive protein (CRP) was associated with a researcher-created composite score for PPD symptoms; however, CRP levels did not significantly predict EPDS scores [129]. Studies by Brann and colleagues and Bianciardi and colleagues failed to find any significant associations between markers of inflammation and PPD [119,120].

Results on maternal epigenetic age and social context displayed an unanticipated direction of the findings in the one included study. In a U.S.-based study using repeated measures of DNA methylation from two separate longitudinal cohorts of pregnant individuals (total N = 229), an unexpected finding that decreased maternal epigenetic age was found to be significantly associated with prenatal stress (PSS score) in the Black/African American subset of one of the cohorts. No significant findings were found among the subset of European/White participants [126].

In a longitudinal cohort study of 150 Latina women in the U.S., Incollingo Rodriguez and colleagues found that telomere length was a significant negative predictor of EPDS score/PPD. In the same study, telomere length was not associated with participants’ experiences of discrimination [124].

## 4. Discussion

This review demonstrates that postpartum mental health outcomes are directly related to the role and behaviors of perinatal health care providers and staff. Specifically, researchers found that experiencing mistreatment and obstetric violence are associated with adverse mental health outcomes, whereas having a supportive maternity care team and birth companion (such as a doula) were associated with more positive mental health outcomes.

Recognizing the important influence of quality of care on maternal and infant birth outcomes, including maternal mental health, the World Health Organization has stated that service users’ experiences of maternity care are an equal contributor to quality-of-care metrics as is the clinical care provided [137,138]. Respectful maternity care is an integral part of high quality care [139] that improves experiences of care and addresses health inequities [140]. Research has demonstrated that marginalized individuals experience care that is less respectful and, relatedly, have higher rates of PMADs [27,28,29,141,142,143,144]. Similarly, a recent systematic review specifically looking at obstetric violence and maternal mental health found a significant association between experiences of obstetric violence and PPD and birth-related PTSD [145]. Conversely, more respectful, person-centered maternity care has been shown to improve birth outcomes [33].

Many of the exposures we evaluated had a significant association with PMADs, and are directly related to the role and behaviors of the personnel on the maternity care team (i.e., mistreatment, obstetric violence, birth experience, support of maternity care team, presence of a birth companion). We postulate that the very process of receiving care for pregnancy and birth may be iatrogenically contributing [146,147] to inequities in maternal mental health, especially for marginalized women and birthing people who report more negative care experiences and mistreatment and higher rates of PMADs.

This review did not identify studies reporting significant associations between discrimination and PMADs. It is possible that individuals who experienced discrimination from clinicians during perinatal care would be less likely to continue engagement with the healthcare system or research in the postpartum period [148,149] due to a decreased trust of their healthcare team [150]. Decreased engagement would lead to lower rates of postpartum screening and diagnosis of PMADs, as well as other significant postpartum morbidities, for individuals exposed to discrimination.

There is increasing evidence that biological changes related to social context, stress, and discrimination may contribute to negative birth outcomes [151,152,153,154,155,156]. In this review, we identified only a small number of studies that specifically explored the relationship between biological markers of weathering and PMADs. Studies varied widely in sample size, study design, biological markers measured and had inconsistent findings. Further research should explore associations between biological indicators of weathering and a holistic set of biopsychosocial birth outcomes, including perinatal mental health. This research is most needed among marginalized women and childbearing people who have higher rates of mistreatment, discrimination, and mental health complications, and are at greater risk for adverse postpartum and neonatal outcomes. Given the potential for negative experiences to affect multiple generations through fetal intrauterine programming in the prenatal period [157], this research is critical for mitigating health inequities across multiple generations.

*Limitations.* Although the search strategy for this review was comprehensive and systematic, it is possible that it excluded some studies that may have strengthened or disconfirmed the study findings. It is possible that publication bias reduced the number of null findings in existent literature, given that many of the studies that included biomarkers reported null or weakly significant findings. An additional limitation of this review is that studies did not generally include individuals with preexisting mental health diagnoses, which are a known risk factor for PMADs. Therefore, results from this review cannot be generalized to this high-risk group. Finally, we recognize that there are many forms of marginalization based on gender, sexual orientation, language/immigration status, rural residence, and intersections thereof that may also affect postpartum mental health that were not explored in this review.

*Implications for practice*, *policy*, *and research*. Our findings have highlighted areas of the social context of perinatal care that can be improved to potentially decrease the incidence of PMADs and associated morbidity and mortality. This research can be used to inform social policy and develop clinical interventions to decrease iatrogenic influences on PMADs. It may also guide patient-centered research to improve perinatal care experiences, as well as the development of interventions and social policies to alleviate stress and improve socioeconomic standing for those at highest risk of PMADs. Further research into mutable social influences on perinatal mental health and their biologic underpinnings can help to design and tailor targeted effective treatments and prevention measures, especially for those most at risk for negative outcomes.

Clinical practice changes that improve the experience of the clinician-service user interaction may directly influence the iatrogenic influences on PMADS. By further emphasizing techniques that are well-evidenced in the literature, such as providing respectful, person-centered care to all patients, improving provider-patient communication, increasing their use of shared decision making practices, and supporting patients’ use of doulas or birth companions, providers may alleviate stress associated with interactions with the maternity healthcare team or service users’ perceptions of mistreatment [141,158,159,160]. Providing additional supports for persons experiencing socioeconomic stressors by increasing tailored referrals to supportive services (i.e., food and housing supports, diaper banks, social services providing infant-care supplies, social work services, and low- or no-cost doula services) may reduce socioeconomic stressors in the perinatal period that have been found to be associated with increased rates of PMADs in this review.

Combining clinical practice improvements with social policy changes is especially important to consider, given that marginalized women and birthing people who are most likely to experience mistreatment from their healthcare team are also facing an increased incidence of the non-medical social contributors to PMADs identified in this review, such as multiple prenatal stressors and socioeconomic disadvantage. These non-medical social contributors are largely a result of systemic marginalization, structural racism, and oppression [161,162,163,164,165], and warrant large-scale social policy intervention to mitigate their effects on perinatal mental health and other birth-related outcomes.

## 5. Conclusions

In conclusion, social context and the experience of perinatal care are associated with postpartum mental health outcomes. Individuals who experienced socioeconomic disadvantage and related stressors, and those who experienced mistreatment or obstetric violence, were most likely to experience PMADs. Therefore, increasing delivery of respectful maternity care and broad systemic and structural improvement of socioeconomic circumstances is necessary to reduce postpartum mental health inequities, especially for those made marginal by systemic oppression.

## Figures and Tables

**Table 1:** Description of included studies.

Author (Year) [Citation]	N	Loc	Des	Exp	PMAD	Results
Alcorn (2010) [60]	776	Australia	PL	TB	PTSD ANX PPD	A total of 45.5% of the sample (n = 394) reported a traumatic birth event at 4–6 weeks post-partum. All participants who met full PTSD criteria at 4–6 weeks postpartum had experienced at least one birthing event during which they believed either their life/well-being or the life/well-being of their baby was in danger.
Alhasanat (2017) [61]	50	USA	R	ST	PPD	Among Arab-American WIC recipients, life stress significantly correlated to EPDS score (*p* = 0.02).
Catala (2021) [62]	116	Spain	PL	BES	PPD PTSD	In bivariate analysis, significant association between PTSD and satisfaction with the professionals at labor (*p* = 0.036). 14.6% (n = 17) of sample had EPDS scores indicating probable depression and 7.7% (n = 9) had probable PTSD.
Cena (2021) [63]	1251	Italy	PL	SES	PPD	PPD is associated with participants reporting “several to many” economic problems (*p* < 0.01).
Clout (2015) [64]	105	Australia	PL	ST	PPD	Stressful life events were associated with EPDS score (*p* < 0.05).
Coburn (2016) [65]	269	USA	PL	ST	PPD	Higher levels of daily hassles (*p* = 0.013), partner stress (*p* < 0.001), and family stress (*p* = 0.013) were associated with more postpartum depressive symptoms.
Cruise (2018) [66]	10,827	Ireland	PL	SES	PPD	Tenants were almost twice as likely to be depressed as owner occupiers (OR 1.80; 95% CI 1.59–2.03). Participants in the lowest income quintile were over three times more likely to be depressed as their more affluent peers (OR 3.53; 95% CI 2.81–4.42).
De Schepper (2016) [67]	229	Belgium	PL	TB SES	PTSD	Participants who experienced birth as a traumatic event had a 46% higher likelihood of developing PTSD. Odds of PTSD were 6X more likely in women with an annual family income of less than EUR 2500.
DesMarais (2014) [68]	100	Canada	R	ST IPV	PPD PTSD ANX	Corrected univariate models for IPV before and during pregnancy were significant for stress (*p* < 0.05), anxiety (*p* < 0.01), and posttraumatic stress disorder (*p* < 0.001). The corrected univariate model for IPV during, but not before, pregnancy was additionally significant for depression (*p* < 0.05).
Edge (2007) [69]	200	UK	PL	SES	PPD	Black Caribbean women were twice as likely as White British women to live in the most deprived areas of the city (*p* < 0.001), to be living on benefits (*p* < 0.05), and to be single parents (*p* < 0.05). Black Caribbean women (27%, n = 19) were not more likely than White women (21%, n = 27) (*p* = 0.307) to screen positive for PPD.
Edwards (2008) [70]	154	Australia	PL	ST	PPD	Among socially disadvantaged women in Australia, the only antenatal risk factor found to predict PPD was childhood emotional abuse.
Ertan (2021) [71]	916	France	CS	TB	PTSD PPD	A traumatic experience of birth was associated with higher postpartum EPDS scores.
Farr (2014) [72]	4451	USA	CS	ST SES	ANX PPD	Women with the highest prevalence of postpartum anxiety symptoms experienced 6–13 (16.1%) or 3–5 (15.9%) stressors during pregnancy. Women with the highest prevalence of comorbid postpartum anxiety and depressive symptoms experienced 6–13 stressors during pregnancy (17.3%). Women with the highest prevalence of only postpartum depressive symptoms were those experiencing 6–13 stressors during pregnancy (5.8%) and women with annual household incomes between USD 10,000 and USD 14,999 (4.2%).
Ford (2011) [73]	138	UK	PL	BES	PTSD	At three months postpartum, maternity care staff support was a significant predictor of PTSD symptoms (*p* < 0.05).
Garthus-Niegal (2013) [74]	1499	Norway	PL	BES	PTSD	Birth experiences were significantly associated with post-traumatic stress symptoms.
Handelzalts (2022) [75]	246	Israel	PL	BC	PTSD	Women who were accompanied by their partners and an additional companion were lower in birth-related PTSD symptoms than women accompanied by only their partner (*p* < 0.05).
Handelzalts (2022) [76]	254	Israel	PL	BES	PTSD PPD	Perception of birth experience (*p* < 0.05) significantly predicted birth-related PTSD (BiTS) in linear regression models. No significant findings for birth experience and PPD.
Harrison (2021) [77]	16,000	UK	CS	BES	PTSD	Factors significantly associated with birth related PTSD: higher level of deprivation, not having a health care professional to talk to about sensitive issues during pregnancy, having an instrumental or caesarean birth, experiencing childbirth as worse than expected.
Hein (2014) [78]	1100	Germany	PL	SES	PPD	Having a low income, renting home vs. owning, and less than high school education were all significantly associated with higher EPDS scores, but few participants screened positive for PPD.
Hernández-Martínez (2020) [79]	1531	Spain	CS	RMC	PTSD	The presence of traumatic stress symptoms was identified in 7.2% of the study population. Variables found to be protective factors against PTSD symptoms included having the birth plan respected (aOR 0.44; 95% CI 0.24–0.80).
Holt (2018) [80]	1393	UK	R	BES	PTSD PPD	Fearful birth experiences and traumatic birth appraisals were both significantly correlated with PTSD symptoms and PPD.
Janssen (2012) [81]	6421	Canada	CS	IPV SES	PPD	Among abused women: age extremes were associated with PPD. Among non-abused women: unemployment (aOR 1.41; 95% CI 1.06–1.84), foreign birth (aOR 2.04; 95% CI 1.35–3.09], and low income (aOR 1.68; 95% CI 1.25–2.25) were associated with PPD. PPD was significantly associated with abuse occurring only prior to pregnancy (aOR 3.28; 95% CI 1.86–5.81), starting postpartum (aOR 4.76; 95% CI 1.41–16.02), and resuming postpartum (aOR 3.81; 95% CI 1.22–11.88).
Jones (2018) [82]	295	USA	R	SES	PPD	Residential stability was found to be statistically significantly associated with PPD symptom severity based on the 3-month EPDS total score (*p* = 0.01). Social disadvantage was not found to be significant (*p* = 0.12).
Katon (2014) [83]	1423	USA	PL	ST	PPD	In a cohort of high-risk prenatal clinic patients, the total stress score was significantly associated with PPD [OR 1.14; 95% CI 1.09–1.19].
Kim (2012) [84]	838	USA	CS	SES	PPD	Postpartum depression symptoms were present in 17% (n = 55), and were associated with being single (aOR 2.41; 95% CI 1.29–4.50), first time mother status (aOR 2.43; 95% CI 1.34–4.40), and living in temporary housing (aOR 2.35; 95% CI 1.30–4.26).
Kjerulff (2021) [85]	3006	USA	PL	BES ST	PTSD	Scores on the FBS Birth Experience Scale strongly associated with childbirth-related PTSD (CR-PTSD). Women who reported CR-PTSD symptoms were less positive about their childbirth experience than women who did not experience CR-PTSD. Shared decision-making (SDM) was also strongly associated with CR-PTSD. Women who reported experiencing CR-PTSD reported lower levels of SDM than women who did not experience CR-PTSD. Women with low social support and high stress during the third trimester before childbirth were more likely to report CR-PTSD symptoms.
Kothari (2016) [86]	301	USA	CS	SES IPV	PPD	Poor participants had significantly higher postpartum depression scores than nonpoor participants (mean EPDS 6.0 and 4.7, respectively, *p* = 0.017). IPV and poverty were positively associated with each other (*p* < 0.001) and with EPDS score (IPV: *p* < 0.001; poverty: *p* = 0.017). In multiple linear regression, IPV remained significantly associated, but poverty did not (IPV: adjusted *p* < 0.001; poverty: adjusted *p* = 0.141).
Kress (2021) [87]	1146	Germany	C	TB	PTSD PPD	Poorer subjective birth experience predicted PTSD symptoms with small to moderately sized effects. Level of support during birth was moderately associated with PTSD symptoms (low support associated with higher PTSD symptoms).
LaCoursiere (2012) [88]	1054	USA	R	ST	PPD	Women reporting a physical fight during pregnancy had a fourfold increased odds of screening positive for PPD (OR = 4.09; 95% CI 1.23–13.54). As the frequency of stressors increased, the prevalence of screening positive for PPD also increased. Approximately 10% (30 of 293) of women without any stressors screened positive for PPD, 15.4% (62 of 402) of women with one stressor category, 17.2% (39 of 188) with two stressor categories, 41.3% (31 of 75) with three different categories of stressors, and 61.9% (13 of 21) of women reporting stressors in all four categories (Chi-square test for trend *p* < 0.001).
Leavy (2023) [89]	123	France	C	MIST	PTSD PPD	Reported disrespect during childbirth was significantly associated with higher childbirth-related PTSD (CB-PTSD) 2 months after birth (*p* < 0.001). PPD at 2 months after childbirth was positively associated with reported disrespect in the birth room (*p* = 0.01). PPD and CB-PTSD were significantly associated 2 months after childbirth (*p* < 0.01).
Liu (2018) [90]	3010	USA	R	ST	PPD	Factors associated with PPD included having at least one stressor, with a 1.8 X increase with 1–2 stressors endorsed, 3.4 X increase with 3–5 stressors, and 10.6 X increase with 6+ stressors.
Luecken (2019) [91]	322	USA	PL	ST	PPD	Depressive symptoms at 24 weeks postpartum were associated with prenatal economic hardship and prenatal stressful life events.
Luis Sanchez (2020) [92]	159	USA	PL	DISC ST	PPD	Increases in general perceived stress symptoms from pregnancy to postpartum contributed to depressive symptoms postpartum.
Mallicoat (2020) [59]	4245	UK	C	SES	Maternal Mental Health	In a cohort of women of Pakistani ethnicity, three different SES measures showed statistically significant relationships with mental health (all *p* < 0.0005).
Martinez-Vázquez (2022) [93]	782	Spain	CS	OV	PPD	Risk factors for PPD included experiencing verbal obstetric violence (aOR 2.02; 95% CI 1.35–3.02), and psycho-affective obstetric violence (aOR 2.65; 95% CI 1.79–3.93). The perception of support during pregnancy, birth, and postpartum was protective against PPD (aOR 0.15; 95% CI 0.04–0.54) for women who perceived “enough support” and further protective for women who received “much support” (aOR 0.13; 95% CI 0.0–0.45).
Mukherjee (2017a) [17]	115,704	USA	R	ST	PPD	The highest prevalence of postpartum depression was in the multiple stress class, followed by illness/death, and low-stress classes.
Mukherjee (2017b) [94]	91,253	USA	R	SES	PPD	Women who experienced all four stressor categories, including partner-related, traumatic, emotional, and financial, had the highest odds of PPD symptoms (aOR 5.43; 95% CI 5.36–5.51). The odds of experiencing PPD symptoms decreased with an increase in the state-level social/economic autonomy index (aOR 0.75; 95% CI 0.64–0.88). There was significant cross-level interaction between number of stressor categories experienced and state-level SES index.
Mukherjee (2018) [95]	87,565	USA	R	ST SES	PPD	Women in the lower income and education categories generally had a higher prevalence of PPD than those in the highest categories. Financial stress was a significant risk factor for PPD. Those who experienced a stressful life event had a higher unadjusted prevalence of PPD than those that did not experience it.
Nakamura (2020) [96]	14,587	France	C	SES	PPD	SES was negatively associated with EPDS score, with an increase of one unit of SES associated with a reduction of, respectively, 6%, 10%, and 16% of EPDS score in non-migrant women (RR = 0.94; 95% CI 0.91–0.96), second generation migrant women (RR = 0.90; 95% CI 0.86–0.96) and first-generation migrant women (RR = 0.84; 95% CI 0.76–0.95).
Nakic Rados (2021) [97]	603	Croatia	CS	BES	PTSD PPD	In an online study of women within 12 months postpartum, low birth satisfaction (including stress during labor and quality of care provided) was associated with higher PTSD symptoms, but not PPD symptoms.
Nunes (2013) [98]	6283	USA	R	ST	PPD	Among mothers over the age of 25, stressors were associated with increased odds of reporting PPD symptoms. The strongest risk factors among the stressor variables included “argue more than usual”, “physical fight”, “couldn’t pay bills”, “use of drugs by others”, and “partner did not want pregnancy”. Across all age ranges, combined stress score was strongly associated with PPD symptoms. Participants reporting six or more stressors had over 20X odds of reporting PPD symptoms as compared to those reporting no stressors.
O’Donovan (2014) [99]	933	Australia	PL	TB	PTSD	Of the 45.5% of women who reported that their birth experience was traumatic (n = 394), 7.9% developed PTSD between 4 and 6 weeks postpartum (n = 31). Primagravidas were more likely to report that their birth was traumatic. Event-related psychological variables that predicted birth trauma were perceived lack of control during labor, low self-efficacy, discrepancy in expectations around the birthing event, and feeling unprepared for the birth.
Ogbo (2019) [100]	25,407	Australia	R	SES	PPD	Higher SES had a protective effect on PPD symptoms.
Paiz (2022) [101]	287	Brazil	CS	MIST SES	PPD	Women who experienced mistreatment during childbirth had a higher prevalence of symptoms suggestive of PPD (PR 1.55; 95% CI 1.07–2.25). Higher socioeconomic status had an inverse association with PPD (PR 0.53; 95% CI 0.33–0.83).
Pinheiro (2012) [102]	276	Brazil	CS	ST	PPD	EPDS score > 13 was associated with the presence of SLE (*p* < 0.01).
Price (2009) [103]	1086	USA	R	SES	PPD	Significantly increased odds of PPD when eligible for Temporary Aid for Needy Families (TANF; signifier of low SES).
Qobadi (2016) [104]	3695	USA	R	ST	PPD	Mothers who experienced high relational stress, low financial stress and high trauma-related stresses had the highest likelihood of PPD diagnosis after adjusting for confounders (aOR 8.6; 95% CI 3.5–21.3), followed by those who reported high relational stress with low financial and trauma-related stress (OR 5.9; 95% CI 3.5–10.2) compared to women with low stress in all three categories.
Razurel (2017) [105]	235	Switzerland	PL	ST	PPD ANX	In a mainly high-income, well-educated sample, health professional support immediately post-birth displayed a significant interaction with mental health outcomes. Perceived stress was significantly associated with EPDS scores.
Roberts (2022) [106]	392	Ireland	PL	TB	PTSD ANX PPD	For the 6 month postpartum EPDS, prenatal EPDS, any maternal mental health history, anxiety screening at 6 months, and experience of traumatic birth explained 56.2% of variability (*p* < 0.001).
Salm Ward (2017) [107]	10,231	USA	R	ST	PPD	Significantly higher odds of reporting PPD symptoms were identified among mothers with less than a high school education (compared to college graduates, OR 1.70; 95% CI 1.13–2.54), living in a rural area (versus urban, OR 1.28; 95% CI 1.03–1.60), using Medicaid for delivery (versus private insurance, OR 1.45; 95% CI 1.07–1.95), with an unintended pregnancy (versus intended, OR 1.78; 95% CI 1.31–2.41). As the number of cumulative SLEs increased, the odds of reporting PPD symptoms also increased, with the greatest odds of reporting PPD symptoms among mothers who have experienced six or more SLEs (OR 5.77; 95% CI 3.89–8.55). After controlling for significant sociodemographic variables, each SLE was still associated with increased odds of PPD symptoms.
Silveira (2019) [108]	3065	Brazil	C	MIST	PPD	Verbal abuse from maternity care personnel increased odds of having moderate PPD (OR 1.58; 95% CI 1.06–2.33) and severe PPD (OR 1.69; 95% CI 1.06–2.70). Physical abuse increased the odds of having marked/severe PPD (OR 2.28; 95% CI 1.26–4.12). Having experienced three or more mistreatment types increased the odds of moderate PPD (OR 2.90; 95% CI 1.30–35.74) and severe PPD (OR 3.86; 95% CI 1.58–9.42).
Sommerlad (2021) [109]	284	Germany	PL	BES	PTSD	A positive birth experience directly reduced CB-PTSD symptoms (*p* < 0.01).
Souza (2017) [110]	10,468	Brazil	CS	OV	PPD	Experiencing obstetric violence was an independent predictor of postpartum Edinburgh Postnatal Depression Scale scores.
Steetskamp (2022) [111]	278	Germany	PL	BC	PTSD	A total of 6.3% (13/206) of those without a companion during labor had PTSD symptoms vs. 1% (4/383) of those who had a companion (*p* < 0.001). PTSD was seen more often in patients with a migrant background (*p* = 0.007). Maternal age (*p* < 0.001), parity (*p* < 0.001), migrant background (*p* < 0.001), assistance during labor (*p* < 0.001) and the mode of delivery (*p* = 0.001) influence PTSD symptom severity.
Stone (2015) [112]	5395	USA	R	ST	PPD	Reporting of one or more stressors was associated with increased prevalence of PPD (PR 1.68; 95% CI 1.42–1.98). The strongest association was observed for partner stress (PR 1.90; 95% CI 1.51–2.38).
Suarez (2023) [113]	2579	Russia	CS	TB BC	PTSD PPD	A total of 37.5% of participants had clinically significant depressive symptoms (EPDS scores > 10). In total, 20.5% of women fulfilled all the DSM-5 diagnostic criteria for PTSD (according to CBiTS scores). Both PPD (Pearson correlation = 0.34, *p* < 0.001) and PTSD (Pearson correlation = 0.46, *p* < 0.001) significantly correlated with the subjective birth trauma. Women who gave birth in the presence of a support person scored lower on both the postpartum PTSD scale and the subjective scale of traumatic birth experience and had a significantly lower risk of having a clinical postpartum-PTSD diagnosis.
Tebeka (2021) [114]	3310	France	PL	ST	PPD	Early and late onset PPD were significantly associated with stressful life events in pregnancy (early onset OR 2.0; 95% CI 1.5–2.7; late onset OR 2.4, 95% CI 1.8–3.2).
Waller (2022) [115]	1082	USA	PL	TB	PPD	Patient-reported childbirth trauma is significantly associated with postpartum depression (OR 1.33; 95% CI 1.10–1.60).
Wikman (2020) [116]	2466	Sweden	R	BES	PPD	Participants with a self-reported negative experience with delivery had 4.3X the likelihood of early postpartum depression (OR 4.3, 95% CI 2.9–6.4).
Yakupova (2022) [117]	611	Russia	CS	OV BC	PTSD PPD	Postpartum PTSD symptoms were higher among women who experienced obstetric violence (*p* < 0.001) during childbirth. The more interventions they had (*p* = 0.012), and the more instances of obstetric violence they experienced (*p* < 0.001), the higher the PTSD symptoms were. The presence of a partner or a personal midwife/doula at birth was associated with lower rates of cesarean birth, fewer medical interventions, and less obstetric violence (*p* < 0.017 for all).

**Location (Loc); Design (Des):** PL: prospective/longitudinal, R: retrospective, C: cohort, CS: cross-sectional; **Experience/Exposure (Exp):** BC: birth companion, BES: birth experience/satisfaction, DISC: discrimination, IPV: intimate partner violence, MIST: mistreatment, OV: obstetric violence, SES: Socioeconomic disadvantage/deprivation, ST: stress, TB: traumatic birth; **PMADs**: ANX: anxiety, PPD: postpartum depression, PTSD: birth-related post-traumatic stress disorder (PTSD); **Measures:** CBTS: City Birth Trauma Scale, EPDS: Edinburgh Postnatal Depression Scale, IES: Impact of Event Scale, STAI: State-Trait Anxiety Inventory; **Other:** SLE: stressful life events, OR: odds ratio, aOR: adjusted odds ratio, CI: confidence interval, RR: relative risk.

**Table 2:** Description of included studies with biological measures.

Author (Year) [Citation]	N	Loc	Des	Exp	BioM	PMAD	Results
Adynski (2019) [118]	845	USA	PL	ST SES	AL	PPD ANX	Odds of elevated depressive symptoms in women with food insecurity were 2.12 (95% CI 1.33–3.37) compared to those without perceived food insecurity. Allostatic load score (OR 0.98; 95% CI 0.91–1.05) and the remaining demographic and social determinants of health predictor variables were not significantly predictive of elevated depressive symptomology.
Bianciardi (2021) [119]	79	Italy	PL	-	INF	PPD	The EPDS total score of the two groups with PPD (with and without trauma) was the dependent variable and the biological markers (cortisol, IL-6, TNF-α) were the independent variables. The results were not significant. The TNF-α OR was 1.856 (*p* = 0.053).
Brann (2017) [120]	291	Sweden	C	-	INF	PPD	The sole significant value on Bonferroni correction was higher IL-10 in controls than those with PPD (*p* = 0.029); this did not hold in sensitivity analyses where those with depression in pregnancy were excluded.
Buglione-Corbett (2018) [121]	110	USA	PL	-	INF	PPD	Elevated serum TNF-α was associated with lower EPDS total score (*p* = 0.046) after adjusting for demographics and medication use. In contrast, IL-6, CRP, and IL-1β did not demonstrate statistically significant associations with depressive symptoms by the EPDS in either crude or adjusted models.
Caparros-Gonzalez (2017) [122]	44	Spain	PL	ST	CORT	PPD	Hair cortisol at the first trimester (*p* < 0.05) and third trimester (*p* < 0.05) significantly predicted EPDS scores. The group with postpartum depression symptoms had higher hair cortisol levels during the first, second, and third trimesters.
Corwin (2015) [123]	152	USA	PL	-	INF	PPD	TNFα levels were significantly different between groups (those symptomatic of PPD and those without PPD) with lower TNFα levels (*p* < 0.05) at all time points in women symptomatic of PPD. There were no differences in any other cytokine or in the ratios of any pro- to anti-inflammatory cytokine among women who did or did not score symptomatic of PPD.
Incollingo Rodriguez (2022) [124]	150	USA	PL	DISC	TL	PPD	TL was a significant negative predictor of postpartum EPDS scores (*p* = 0.024). There were no significant differences in TL based on ethnicity (US born or non-US born), spoken language (English or Spanish), income, welfare status, education, or marital status (all *p*’s > 0.110). The Everyday Discrimination Scale score did not predict TL (*p* = 0.208).
Katrinli (2023) [125]	89	USA	CS	ST	EA	-	Exposure to past-year stressful life events was significantly associated with accelerated epigenetic age in mothers.
Lancaster (2021) [126]	229	USA	PL	ST	EA	-	In the African American/Black subset only, early pregnancy EA [using Horvath’s clock calculator] was inversely related to early perinatal Perceived Stress Scale score. European/White participants did not have significant findings.
Nakamura (2019) [127]	36	Japan	PL	-	DM	PPD	The difference in methylation frequency between the postpartum non-depressed group and the postpartum depressed group was small, and sites with genome-wide significant differences were not confirmed.
Ono (2023) [128]	490	Japan	PL	-	INF	PPD	IL-4 and IL-10 higher during pregnancy in controls than +PPD. All others null.
Roomruangwong (2017) [129]	71	Thailand	PL	-	INF	PPD ANX	↑ CRP: ↑ STAI score (anx), (*p* = 0.003) ↑ CRP: ↑ Multivariable outcome (postnatal depressive symptoms on EPDS, BDI, STAI, Hamilton) (*p* = 0.001). Postnatal EDPS was not predicted by CRP.
Simpson (2016) [130]	33	Canada	PL	-	INF	PPD	IL-6 (*p* = 0.025), and IL-10 (*p* = 0.006) were significant predictors of postpartum EPDS score.
Stickel (2021) [131]	196	Germany	PL	ST	CORT	PPD	Neither SLE nor CORT was associated with PPD.

**Location (Loc); Design (Des):** PL: prospective/longitudinal, R: Retrospective, C: cohort, CS: cross-sectional; **Experience/Exposure (Exp):** DISC: discrimination, SES: Socioeconomic disadvantage/deprivation, ST: stress; **Biomarkers (BioM):** AL: allostatic load, CORT: hair cortisol, DM: DNA methylation, EA: epigenetic age, INF: inflammatory cytokines, TL: telomere length; **PMADs**: ANX: anxiety, PPD: postpartum depression; **Measures:** CBTS: City Birth Trauma Scale, EPDS: Edinburgh Postnatal Depression Scale, IES: Impact of Event Scale, STAI: State-Trait Anxiety Inventory; **Other:** OR: odds ratio, aOR: adjusted odds ratio, CI: confidence interval.

**Table 3:** Instruments to measure exposures in included studies.

Exposure	Measurement Tool	Included Studies Using Measurement Tool
Perinatal stress/stressors	Perceived Stress Scale (PSS)	[118,122,123,126]
	PRAMS questions about stressors	[17,72,88,90,94,95,98,104,107,112]
	Economic Hardship Scale	[91]
	Antenatal Perceived Stress Inventory (APSI)	[105]
	Postpartum Depression Predictors Inventory (PDPI)-Revised	[61]
	Paykel Scale	[114]
	Prenatal Psychosocial Profile Stress Scale	[83]
	Symptoms Screener for Adults (STRESS-A) Turner Life Events Scale	[125]
Discrimination	Everyday Discrimination Scale (EDS)	[124]
	Discrimination Stress Scale	[92]
Birth experiences/satisfaction	Childbirth Experience Questionnaire	[75,76]
	Birth Satisfaction Scale-Revised (BSS-R)	[97]
	Wijma Delivery Experience Questionnaire	[80]
	Women’s View of Birth Labor Satisfaction Questionnaire (WOMBLSQ)	[62]
	First Baby Study Birth Experiences Scale	[85]
	Birth Expectation and Experience Scale	[99]
Interactions with the maternity care team	Support and Control in Birth (SCIB) Questionnaire	[73]
Mistreatment/disrespect during childbirth	Behavior of the Mother’s Caregivers–Satisfaction Questionnaire (BMC-SQ)	[89]

## Data Availability

No new data were created.

## Supplementary Materials

The following supporting information can be downloaded at: https://www.mdpi.com/article/10.3390/ijerph21040480/s1, Table S1: Locations of included studies; Full search strategy. Link to review protocol registration at OSF.
