# Supplementary material for: The Social Context of Pregnancy, Respectful Maternity Care, Biomarkers of Weathering, and Postpartum Mental Health Inequities: A Scoping Review
Source: Int J Environ Res Public Health. 2024 Apr 15;21(4):480. doi: 10.3390/ijerph21040480 (PMC11049830; doi:10.3390/ijerph21040480)
Supplement: Supplementary file 1 [file ijerph-21-00480-s001.zip › ijerph-2935727-supplementary.pdf]

**Supplemental Table S1: Locations of included studies**

| **Region** | **N** | **Country** | **n** |
|---|---|---|---|
| Americas | 34 | Brazil | 4 |
| | | Canada | 3 |
| | | United States | 27 |
| Asia | 7 | Israel | 2 |
| | | Japan* | 2 |
| | | Russia | 2 |
| | | Thailand* | 1 |
| Europe | 28 | Belgium | 1 |
| | | Croatia | 1 |
| | | France | 4 |
| | | Germany | 5 |
| | | Ireland | 2 |
| | | Italy | 2 |
| | | Norway | 1 |
| | | Spain | 4 |
| | | Sweden | 2 |
| | | Switzerland | 1 |
| | | United Kingdom | 5 |
| Oceania | 5 | Australia | 5 |
| Total | 74 | | |
| *Japan and Thailand were included for biomarker studies only | | | |

SUPPLEMENTAL FILE

OSF search protocol registration:

https://osf.io/t5pv7/?view_only=d16279c74d814975a44ac70eef3af2ad

Ovid MEDLINE(R) ALL

| | |
|---|---|
| 1 | [CONCEPT ONE: experiences/exposures/trauma/mistreatment/stress/disadvantage] |
| 2 | ["obstetric violence" interpreted here as being caused by medical personnel] |
| 3 | exp maternal health services/ |
| 4 | exp labor, obstetric/ |
| 5 | exp delivery, obstetric/ |
| 6 | 3 or 4 or 5 |
| 7 | exp violence/or violen$.ti. |
| 8 | 6 and 7 |
| 9 | (obstetric* adj3 violen*).tw,kf. |
| 10 | 8 or 9 |
| 11 | [end of obstetric violence. this term is used more away from USA, and with disadvantaged and immigrants, etc.] |
| 12 | [begin respectful maternity care] |
| 13 | "respectful matern* care".tw,kf. |
| 14 | exp Maternal Health Services/or exp Delivery, Obstetric/or exp Parturition/ |
| 15 | 14 and (respectful$ or polite$ or considerate or dignified or gracious or thoughtful or attentive or mindful).ti,kf. |
| 16 | 13 or 15 |
| 17 | [end respectful. this cd be broadened by finding other words for respectful] |
| 18 | [perinatal stress is an "unfortunate" term because we don't know to whom it applies, mom or baby. the phrase is not used much. but I even saw "paternal perinatal stress" - and then: "stress" is "psychological" for the mom, and metabolic for the baby, says me; very squishy topic] |
| 19 | perinatal stress*.tw. or perinatal stress*.kf. |
| 20 | [end of perinatal stress until we define it better; get a brainstorm; etc.] |
| 21 | [traumatic birth, another phrase not used much. do we mean birth injuries, obstetric complications, or something more psychological?] |
| 22 | exp Delivery, Obstetric/or exp Obstetric Labor Complications/ |
| 23 | 22 and traumatic.ti. |
| 24 | (birth adj3 trauma$).tw,kf. |
| 25 | 23 or 24 |
| 26 | [end of traumatic birth; this could, of course, be broadened, if "traumatic" were to get all fleshed-out] |
| 27 | [pregnancy mistreatment. ouch, another!] |
| 28 | ((mistreat* or cruelt$ or oppression or harassment or exploitation or neglect* or discrimination or brutality or bully* or inequity or malpractice) adj3 pregnan*).tw,kf. |
| 29 | pregnancy mistreatment.af. |
| 30 | [right. "pregnancy mistreatment" is not a thing (in the published medical literature sense of the word)] |
| 31 | [end of pregnancy mistreatment] |
| 32 | [pregnancy marginalization; it's "not a thing" as a phrase; see next set. Of course, as a concept, it's searchable...] |
| 33 | (marginaliz* adj3 pregnan*).tw,kf. |

| | |
|---|---|
| 34 | exp maternal health services/or exp pregnant women/or exp prenatal care/ |
| 35 | exp Social Marginalization/or marginali*.ti. |
| 36 | 34 and 35 |
| 37 | 33 or 36 |
| 38 | [end of marginalization. of course this idea of "marginalizing" could be broadened if defined in more medical-scientific terms...] |
| 39 | [pregnancy & socioeconomic disadvantage or socio... deprivation] |
| 40 | [... and we'll add in "childbirth" (aka parturition)] |
| 41 | exp Socioeconomic Factors/ |
| 42 | (socioeconomic adj2 (factor* or disadvantage* or depriv*)).tw,kf. |
| 43 | 41 or 42 |
| 44 | exp pregnancy/ |
| 45 | exp parturition/ |
| 46 | (pregnancy or childbirth).tw,kf. |
| 47 | 44 or 45 or 46 |
| 48 | 43 and 47 |
| 49 | [end of pregnancy (or childbirth) and socioeconomic factors] |
| 50 | 10 or 16 or 19 or 25 or 37 or 48 |
| 51 | [END OF CONCEPT ONE, WHICH IS EXPERIENCES & EXPOSURES] |
| 52 | [CONCEPT TWO -- BIOMARKERS] |
| 53 | [begin epigenetic age] |
| 54 | (exp Epigenesis, Genetic/or exp epigenomics/) and age.ti. |
| 55 | epigenetic age.tw,kf. |
| 56 | 54 or 55 |
| 57 | [end of epigenetic age] |
| 58 | [begin weathering & allostatic load] |
| 59 | weathering.ti. |
| 60 | "wear and tear".tw,kf. |
| 61 | "deteriorat* health".tw,kf. |
| 62 | (exp allostasis/and load.ti.) or "allostatic load".tw,kf. |
| 63 | 59 or 60 or 61 or 62 |
| 64 | 63 and (exp *pregnancy/or exp *pregnancy complications/or exp *pregnancy outcome/) |
| 65 | [end weathering & allostatic load] |
| 66 | [begin hair cortisol] |
| 67 | (hair adj5 cortisol).af. |
| 68 | exp *Hydrocortisone/and exp *Hair/ |
| 69 | 67 or 68 |
| 70 | [end hair cortisol] |
| 71 | [begin microbiome] |
| 72 | exp Microbiota/ |
| 73 | (microbiota or microbiome).tw,kf. |
| 74 | 72 or 73 |
| 75 | [end microbiome] |

| | |
|---|---|
| 76 | [begin inflammation] |
| 77 | exp Inflammation Mediators/or exp Inflammation/ |
| 78 | inflammat*.ti. or exp allostasis/or exp dna methylation/or exp epigenesis, genetic/or exp epigenomics/or exp hematopoietic stem cells/or exp telomere shortening/or exp gene-environment interaction/or intergenerational transmission.tw,kf. |
| 79 | 77 or 78 |
| 80 | [end of inflammation] |
| 81 | 56 or 64 or 69 or 74 or 79 |
| 82 | [END OF CONCEPT TWO, WHICH IS (selected) BIOMARKERS] |
| 83 | [CONCEPT THREE: BIRTH OUTCOMES FOR MOM AND BABY] |
| 84 | [begin preterm birth] |
| 85 | exp Premature Birth/ |
| 86 | ((premature or preterm) adj2 birth).tw,kf. |
| 87 | 85 or 86 |
| 88 | [end preterm birth] |
| 89 | [begin low birth weight] |
| 90 | exp Infant, Low Birth Weight/ |
| 91 | low birth weight.tw,kf. |
| 92 | 90 or 91 |
| 93 | [end low birth weight] |
| 94 | [begin postpartum depression] |
| 95 | exp Depression, Postpartum/ |
| 96 | postpartum depression.tw,kf. |
| 97 | 95 or 96 |
| 98 | [end postpartum depression] |
| 99 | [begin postpartum anxiety] |
| 100 | (exp Depression, Postpartum/or exp Postpartum Period/) and (exp Anxiety Disorders/or exp Anxiety/) |
| 101 | postpartum anxiety.ti,kf. |
| 102 | 100 or 101 |
| 103 | [end of postpartum anxiety] |
| 104 | [begin PTSD] |
| 105 | exp Stress Disorders, Post-Traumatic/ |
| 106 | ptsd.ti,kf. |
| 107 | post traumatic stress.tw,kf. |
| 108 | 105 or 106 or 107 |
| 109 | [end of PTSD] |
| 110 | [begin severe maternal morbidity] |
| 111 | "severe maternal morbidity".af. |
| 112 | [end severe maternal morbidity] |
| 113 | [begin maternal mortality] |
| 114 | exp maternal mortality/or exp Obstetric Labor Complications/mo [Mortality] |
| 115 | maternal mortality.tw,kf. |
| 116 | 114 or 115 |

| | |
|---|---|
| 117 | [end of maternal mortality] |
| 118 | [begin infant mortality] |
| 119 | exp Infant Mortality/ |
| 120 | infant mortality.tw,kf. or exp pregnancy outcome/ |
| 121 | 119 or 120 |
| 122 | [end infant mortality] |
| 123 | 87 or 92 or 97 or 102 or 108 or 111 or 116 or 121 |
| 124 | [END OF CONCEPT THREE: BIRTH OUTCOMES FOR MOM & BABY] |
| 125 | [COMBINATION OF THREE CONCEPTS] |
| 126 | 50 and 81 and 123 |
| 127 | [NOW, just 2 concepts: experiences/exposures AND Biomarkers] |
| 128 | 50 and 81 |
| 129 | [NOW, just 2 concepts: Biomarkers and Outcomes] |
| 130 | 81 and 123 |
| 131 | [NOW, just 2 concepts: experiences/exposure AND outcomes] |
| 132 | 50 and 123 |
| 133 | 126 or 128 or 130 or 132 |
| 134 | 133 not ((exp animal/or exp invertebrate/or animal experiment/or animal model/or exp plant/or exp fungus/) not exp human/) |
| 135 | limit 134 to english language |
| 136 | [CONCEPT FOUR: MATERNAL MENTAL HEALTH] |
| 137 | exp *Mothers/ |
| 138 | exp *pregnancy/or exp *pregnancy complications/ |
| 139 | exp *Anxiety/ |
| 140 | exp *Stress Disorders, Post-Traumatic/ |
| 141 | exp *depression, postpartum/ |
| 142 | exp *Postpartum Period/and (exp *Anxiety Disorders/or exp *Anxiety/) |
| 143 | (mother$ adj2 "mental health").tw,kf. |
| 144 | maternal mental health.tw,kf. |
| 145 | 137 or 138 |
| 146 | 139 or 140 or 141 or 142 or 143 or 144 |
| 147 | 145 and 146 |
| 148 | 135 and 147 |

OVID Embase

| | |
|---|---|
| 1 | [CONCEPT ONE: experiences/exposures/trauma/mistreatment/stress/disadvantage] |
| 2 | ["obstetric violence" interpreted here as being caused by medical personnel] |
| 3 | exp maternal health services/ |
| 4 | exp labor, obstetric/ |
| 5 | exp delivery, obstetric/ |
| 6 | 3 or 4 or 5 |
| 7 | exp violence/or violen$.ti. |
| 8 | 6 and 7 |
| 9 | (obstetric* adj3 violen*).tw,kf. |
| 10 | 8 or 9 |
| 11 | [end of obstetric violence. this term is used more away from USA, and with disadvantaged and immigrants, etc.] |
| 12 | [begin respectful maternity care] |
| 13 | "respectful matern* care".tw,kf. |
| 14 | exp Maternal Health Services/or exp Delivery, Obstetric/or exp Parturition/ |
| 15 | 14 and (respectful$ or polite$ or considerate or dignified or gracious or thoughtful or attentive or mindful).ti,kf. |
| 16 | 13 or 15 |
| 17 | [end respectful. this cd be broadened by finding other words for respectful] |
| 18 | [perinatal stress is an "unfortunate" term because we don't know to whom it applies, mom or baby. the phrase is not used much. but I even saw "paternal perinatal stress" - and then: "stress" is "psychological" for the mom, and metabolic for the baby, says me; very squishy topic] |
| 19 | perinatal stress*.tw. or perinatal stress*.kf. |
| 20 | [end of perinatal stress until we define it better; get a brainstorm; etc.] |
| 21 | [traumatic birth, another phrase not used much. do we mean birth injuries, obstetric complications, or something more psychological?] |
| 22 | exp Delivery, Obstetric/or exp Obstetric Labor Complications/ |
| 23 | 22 and traumatic.ti. |
| 24 | (birth adj3 trauma$).tw,kf. |
| 25 | 23 or 24 |
| 26 | [end of traumatic birth; this could, of course, be broadened, if "traumatic" were to get all fleshed-out] |
| 27 | [pregnancy mistreatment. ouch, another!] |
| 28 | ((mistreat* or cruelt$ or oppression or harassment or exploitation or neglect* or discrimination or brutality or bully* or inequity or malpractice) adj3 pregnan*).tw,kf. |
| 29 | pregnancy mistreatment.af. |
| 30 | [right. "pregnancy mistreatment" is not a thing (in the published medical literature sense of the word)] |
| 31 | [end of pregnancy mistreatment] |
| 32 | [pregnancy marginalization; it's "not a thing" as a phrase; see next set. Of course, as a concept, it's searchable...] |
| 33 | (marginaliz* adj3 pregnan*).tw,kf. |
| 34 | exp maternal health services/or exp pregnant women/or exp prenatal care/ |
| 35 | exp Social Marginalization/or marginali*.ti. |
| 36 | 34 and 35 |
| 37 | 33 or 36 |

| | |
|---|---|
| 38 | [end of marginalization. of course this idea of "marginalizing" could be broadened if defined in more medical-scientific terms...] |
| 39 | [pregnancy & socioeconomic disadvantage or socio... deprivation] |
| 40 | [... and we'll add in "childbirth" (aka parturition)] |
| 41 | exp Socioeconomic Factors/ |
| 42 | (socioeconomic adj2 (factor* or disadvantage* or depriv*)).tw,kf. |
| 43 | 41 or 42 |
| 44 | exp pregnancy/ |
| 45 | exp parturition/ |
| 46 | (pregnancy or childbirth).tw,kf. |
| 47 | 44 or 45 or 46 |
| 48 | 43 and 47 |
| 49 | [end of pregnancy (or childbirth) and socioeconomic factors] |
| 50 | 10 or 16 or 19 or 25 or 37 or 48 |
| 51 | [END OF CONCEPT ONE, WHICH IS EXPERIENCES & EXPOSURES] |
| 52 | [CONCEPT TWO -- BIOMARKERS] |
| 53 | [begin epigenetic age] |
| 54 | (exp Epigenesis, Genetic/or exp epigenomics/) and age.ti. |
| 55 | epigenetic age.tw,kf. |
| 56 | 54 or 55 |
| 57 | [end of epigenetic age] |
| 58 | [begin weathering & allostatic load] |
| 59 | weathering.ti. |
| 60 | "wear and tear".tw,kf. |
| 61 | "deteriorat* health".tw,kf. |
| 62 | (exp allostasis/and load.ti.) or "allostatic load".tw,kf. |
| 63 | 59 or 60 or 61 or 62 |
| 64 | 63 and (exp *pregnancy/or exp *pregnancy complications/or exp *pregnancy outcome/) |
| 65 | [end weathering & allostatic load] |
| 66 | [begin hair cortisol] |
| 67 | (hair adj5 cortisol).af. |
| 68 | exp *Hydrocortisone/and exp *Hair/ |
| 69 | 67 or 68 |
| 70 | [end hair cortisol] |
| 71 | [begin microbiome] |
| 72 | exp Microbiota/ |
| 73 | (microbiota or microbiome).tw,kf. |
| 74 | 72 or 73 |
| 75 | [end microbiome] |
| 76 | [begin inflammation] |
| 77 | exp Inflammation Mediators/or exp Inflammation/ |
| 78 | inflammat*.ti. or exp allostasis/or exp dna methylation/or exp epigenesis, genetic/or exp epigenomics/or exp hematopoietic stem cells/or exp telomere shortening/or exp gene-environment interaction/or intergenerational transmission.tw,kf. |

| | |
|---|---|
| 79 | 77 or 78 |
| 80 | [end of inflammation] |
| 81 | 56 or 64 or 69 or 74 or 79 |
| 82 | [END OF CONCEPT TWO, WHICH IS (selected) BIOMARKERS] |
| 83 | [CONCEPT THREE: BIRTH OUTCOMES FOR MOM AND BABY] |
| 84 | [begin preterm birth] |
| 85 | exp Premature Birth/ |
| 86 | ((premature or preterm) adj2 birth).tw,kf. |
| 87 | 85 or 86 |
| 88 | [end preterm birth] |
| 89 | [begin low birth weight] |
| 90 | exp Infant, Low Birth Weight/ |
| 91 | low birth weight.tw,kf. |
| 92 | 90 or 91 |
| 93 | [end low birth weight] |
| 94 | [begin postpartum depression] |
| 95 | exp Depression, Postpartum/ |
| 96 | postpartum depression.tw,kf. |
| 97 | 95 or 96 |
| 98 | [end postpartum depression] |
| 99 | [begin postpartum anxiety] |
| 100 | (exp Depression, Postpartum/or exp Postpartum Period/) and (exp Anxiety Disorders/or exp Anxiety/) |
| 101 | postpartum anxiety.ti,kf. |
| 102 | 100 or 101 |
| 103 | [end of postpartum anxiety] |
| 104 | [begin PTSD] |
| 105 | exp Stress Disorders, Post-Traumatic/ |
| 106 | ptsd.ti,kf. |
| 107 | post traumatic stress.tw,kf. |
| 108 | 105 or 106 or 107 |
| 109 | [end of PTSD] |
| 110 | [begin severe maternal morbidity] |
| 111 | "severe maternal morbidity".af. |
| 112 | [end severe maternal morbidity] |
| 113 | [begin maternal mortality] |
| 114 | exp maternal mortality/ |
| 115 | maternal mortality.tw,kf. |
| 116 | 114 or 115 |
| 117 | [end of maternal mortality] |
| 118 | [begin infant mortality] |
| 119 | exp Infant Mortality/ |
| 120 | infant mortality.tw,kf. or exp pregnancy outcome/ |

| | |
|---|---|
| 121 | 119 or 120 |
| 122 | [end infant mortality] |
| 123 | 87 or 92 or 97 or 102 or 108 or 111 or 116 or 121 |
| 124 | [END OF CONCEPT THREE: BIRTH OUTCOMES FOR MOM & BABY] |
| 125 | [COMBINATION OF THREE CONCEPTS] |
| 126 | 50 and 81 and 123 |
| 127 | [NOW, just 2 concepts: experiences/exposures AND Biomarkers] |
| 128 | 50 and 81 |
| 129 | [NOW, just 2 concepts: Biomarkers and Outcomes] |
| 130 | 81 and 123 |
| 131 | [NOW, just 2 concepts: experiences/exposure AND outcomes] |
| 132 | 50 and 123 |
| 133 | 126 or 128 or 130 or 132 |
| 134 | 133 not ((exp animal/or exp invertebrate/or animal experiment/or animal model/or exp plant/or exp fungus/) not exp human/) |
| 135 | limit 134 to english language |
| 136 | [CONCEPT FOUR: MATERNAL MENTAL HEALTH] |
| 137 | exp *Mothers/ |
| 138 | exp *pregnancy/or exp *pregnancy complications/ |
| 139 | exp *Anxiety/ |
| 140 | exp *Stress Disorders, Post-Traumatic/ |
| 141 | exp *depression, postpartum/ |
| 142 | exp *Postpartum Period/and (exp *Anxiety Disorders/or exp *Anxiety/) |
| 143 | (mother$ adj2 "mental health").tw,kf. |
| 144 | maternal mental health.tw,kf. |
| 145 | 137 or 138 |
| 146 | 139 or 140 or 141 or 142 or 143 or 144 |
| 147 | 145 and 146 |
| 148 | 135 and 147 |
